# Exploring the nuances of preterm growth charts

**DOI:** 10.1016/j.jped.2026.101616

**Published:** 2026-09-20

**Authors:** Jae H. Kim

**Affiliations:** University of Cincinnati College of Medicine, Cincinnati Children’s Hospital Medical Center, Cincinnati, OH, USA

Preterm infants face the formidable challenge of adapting early to extrauterine life while sustaining age-appropriate growth and development. Careful nutritional and growth assessment is therefore essential to optimizing their health outcomes. Over recent decades, greater emphasis on measuring, graphing, and reviewing anthropometric data has enabled earlier recognition of growth faltering. This responsiveness is critical because preterm infants have steep growth trajectories, and even small daily deficits in nutrient and energy delivery can accumulate rapidly.

The growth chart serves as a regular reminder of the substantial metabolic needs of preterm infants, who require nutrients beyond those needed for maintenance. Although most healthcare providers have moved from imprecise paper plotting to automated display of percentiles and z-scores in electronic health records (EHRs), using these data to manage growth remains an art. Variability and confusion persist regarding which chart to use at a given gestational age and how to transition between successive charts. Key questions include: Which growth chart is most appropriate for preterm infants? When should clinicians transition to the WHO charts? How well do these charts predict important health outcomes?

Ironically, some challenges in neonatal nutritional management arise because healthcare providers can choose among several excellent growth charts. The Fenton chart integrates large population cohorts, whereas INTERGROWTH was developed as a prescriptive growth standard. Both support monitoring preterm infant growth, but their nuanced differences and areas of overlap complicate the interpretation of patient-level data and changes in growth assessment across charts.

The publication and dissemination of the INTERGROWTH growth standards represented a major advance in neonatal nutrition, similar to the release of the WHO growth charts in 2006, because they were derived from a carefully selected, healthy, multinational cohort [1,2]. Although this dataset might have been expected to provide the definitive standard for assessing growth in preterm infants, the selection process resulted in the near absence of very preterm infants born before 34 weeks’ gestation. Selection criteria emphasizing maternal health and favorable social conditions substantially reduced the occurrence of very preterm birth. The INTERGROWTH team later broadened its criteria to include 408 infants, thereby increasing representation of births before 34 weeks [1]. Nevertheless, the small cohort and greater data variability limit its exclusive clinical use for infants born before 34 weeks’ gestation.

In the current paper, “Agreement between Fenton and INTERGROWTH curves in assessing birth weight of preterm infants,” da Cunha et al. [3] examine differences between the Fenton and INTERGROWTH charts across newborn populations. Their contemporary cohort included 2529 preterm infants, and the analysis focused primarily on weight. Overall agreement between the charts was reasonable (mean bias, 0.08; limits of agreement, 0.67 to 0.50), although INTERGROWTH produced slightly higher z-scores. Differences were driven less by gestational-age strata than by the small-for-gestational-age (SGA) subgroup. Among infants born at 26 to 32 weeks, the Fenton chart assigned higher z-scores in the SGA subgroup and therefore classified fewer infants as SGA, although the greater variability in INTERGROWTH data should be acknowledged. Agreement was better among moderate- to late-preterm infants, for whom INTERGROWTH assigned relatively higher z-scores. The study does not establish a preferred chart; instead, it underscores the continuing need for growth-assessment tools that are easier to interpret and apply consistently in clinical practice.

In 2025, the third iteration of the Fenton growth chart was published [4]. This update was based on a meta-analysis of 4.8 million births from 15 countries, including >174,000 infants born before 30 weeks. The newest Fenton chart advances preterm growth assessment by addressing the previous inclusion of births complicated by impaired fetal growth, such as intrauterine growth restriction. This approach remains distinct from a growth standard and does not account for broader influences of maternal health and socioeconomic conditions on pregnancy and preterm birth. The x-axis was rescaled to exact gestational age in weeks and days rather than completed weeks, improving the precision of weekly tracking. In practice, the largest difference from the 2013 Fenton chart occurs at 28 weeks’ gestation: infants who were at the 29th percentile for girls or the 27th percentile for boys on the older chart are now at the 10th percentile, meaning that more preterm infants will be classified as SGA [5].

The Olsen chart is an older but frequently used population-based preterm growth chart, used primarily in the United States [6,7]. It provides the largest representation of infants born in the United States and, compared with the 2013 Fenton and INTERGROWTH charts, tends to classify more infants as SGA. As a reference chart, it carries population-level bias even with careful selection because it reflects the health profile of the U.S. population, including comparatively high rates of maternal obesity and metabolic syndrome. It is important to note that Olsen chart data are included among the sources used to construct the Fenton chart.

In a concurrent publication, with the support from a Fulbright scholarship, Da cunha (Perna) et al worked in collaboration with 4 sites in Ohio, USA to evaluate the relationship of three growth charts (INTERGROWTH, Fenton (2013) and Olsen) and body composition [8]. In this retrospective cohort of 258 infants, they showed that all three charts concurred well on weight z scores and malnutrition categorization. They showed in all charts that weight z score change and size-for-gestational-age were significantly associated with body composition z scores. However, Fenton identified more infants with low fat-free mass and malnutrition while INTERGROWTH identified more infants with high fat mass and no malnutrition.

Numerous opportunities remain to improve the practical clinical use of growth charts. Recently, the American Academy of Pediatrics published new clinical guidelines replacing the outdated term “failure to thrive” with “growth faltering” [9]. These guidelines also reinforced the use of z-scores rather than percentiles alone, which offer a limited view of the range of growth patterns across infants. Z-scores quantify distance from the median without the constraints of percentile boundaries. Greater use of z-scores should support more precise longitudinal tracking and improved EHR graphics, including plots of z-score over time, thereby facilitating earlier recognition of growth faltering. Another opportunity for improvement is better recognition of how fetal growth trajectories shape postnatal growth expectations, because substantial growth restriction before birth warrants a different interpretation than growth without prenatal restriction. All infants eventually transition from one growth chart to another; as the authors emphasize, the same z-score should not be assumed to have identical meaning across charts.

Further work is needed to define clinically meaningful malnutrition in preterm infants using outcome-based criteria, because many infants experience substantial declines in z-score without evident adverse consequences. Nutritional assessment should therefore synthesize growth-chart data with clinical findings, laboratory and radiographic information, and advanced measures such as body composition. Education is also an important driver of change, and neonatal training should place greater emphasis on applying growth charts in clinical scenarios. Future priorities include comparing the 2025 Fenton charts with INTERGROWTH, conducting more rigorous cross-chart analyses, and standardizing how neonatal providers assess growth and nutritional status.

## Data availability

N/A.

## Conflicts of interest

Grants/Research Support: NIH NICHD Neonatal Research Network, Fresenius-Kabi, Elgan Pharma. Board: NEC Society (unpaid). Consultant: Medela, Alcresta, Infant Biotherapeutics, Mother’s Milk is Best. Shares: Nicolette.
